# Fatigue Damage–Resistant Physical Hydrogel Adhesion

**DOI:** 10.3389/frobt.2021.666343

**Published:** 2021-04-15

**Authors:** Qi Li, Luochang Wang, Qihan Liu, Wei Hong, Canhui Yang

**Affiliations:** ^1^Department of Mechanics and Aerospace Engineering, Southern University of Science and Technology, Shenzhen, China; ^2^State Key Laboratory for Turbulence and Complex Systems, Department of Mechanics and Engineering Science, The Beijing Innovation Center for Engineering Science and Advanced Technology (BIC-ESAT), College of Engineering, Peking University, Beijing, China; ^3^Swanson School of Engineering, University of Pittsburgh, Pittsburgh, PA, United States; ^4^Global Station for Soft Matter, Global Institution for Collaborative Research and Education, Hokkaido University, Sapporo, Japan

**Keywords:** hydrogel, adhesion, fatigue damage resistance, hydrogen bond, wrinkle

## Abstract

Strong adhesion between hydrogels and various engineering surfaces has been achieved; yet, achieving fatigue-resistant hydrogel adhesion remains challenging. Here, we examine the fatigue of a specific type of hydrogel adhesion enabled by hydrogen bonds and wrinkling and show that the physical interactions–based hydrogel adhesion can resist fatigue damage. We synthesize polyacrylamide hydrogel as the adherend and poly(acrylic acid-*co*-acrylamide) hydrogel as the adhesive. The adherend and the adhesive interact *via* hydrogen bonds. We further introduce wrinkles at the interface by biaxially prestretching and then releasing the adherends and perform butt-joint tests to probe the adhesion performance. Experimental results reveal that the samples with a wrinkled interface resist fatigue damage, while the samples with a flat interface fail in ~9,000 cycles at stress levels of 70 and 63% peak stresses in static failure. The endurance limit of the wrinkled-interface samples is comparable to the peak stress of the flat-interface samples. Moreover, we find that the nearly perfectly elastic polyacrylamide hydrogel also suffers fatigue damage, which limits the fatigue life of the wrinkled-interface samples. When cohesive failure ensues, the evolutions of the elastic modulus of wrinkled-interface samples and hydrogel bulk, both in satisfactory agreements with the predictions of damage accumulation theory, are alike. We observe similar behaviors in different material systems with polyacrylamide hydrogels with different water contents. This work proves that physical interactions can be engaged in engineering fatigue-resistant adhesion between soft materials such as hydrogels.

## Introduction

Hydrogels are aggregates of polymer networks and water. The polymer network deforms and maintains shape and the water dissolves small molecules and enables their transportation. The unique combination of solid and liquid properties of hydrogels has enabled their use in enormous applications such as contact lenses (Wichterle and Lim, 1960), superabsorbents (Dubrovskii et al., 1990), cell culture (Thiele et al., 2014), drug delivery (Li and Mooney, 2016), and tissue engineering (Nam and Mooney, 2021). While conventional hydrogels are weak and fragile, the significant progress made in the past two decades or so has led to the creation of hydrogels that are as strong and tough as natural rubbers (Gong et al., 2003; Sun et al., 2012), drastically proliferating the applications of hydrogel. For example, hydrogels infused with mobile ions are featured as stretchable, transparent, ionic conductors that can be used for artificial muscle (Keplinger et al., 2013; Acome et al., 2018), artificial skin (Sun et al., 2014), artificial axon (Yang et al., 2015), artificial eel (Schroeder et al., 2017), touchpad (Kim et al., 2016), liquid crystal device (Yang et al., 2017), triboelectronic generator (Pu et al., 2017), and ionotronic luminescent device (Larson et al., 2016; Yang et al., 2016, 2020), a family of emerging soft devices called hydrogel ionotronics (Yang and Suo, 2018). Other examples include water-matrix composite (King et al., 2015), optical waveguide (Choi et al., 2013), soft robot (Lee et al., 2020; Li Q. et al., 2020), and soft machine (Calvert, 2009; Liu X. et al., 2020).

Practical deployments of hydrogel have been impeded by two long-standing challenges: fatigue and adhesion. As for fatigue, many applications require hydrogels to sustain prolonged static or cyclic loads, whereas most existing hydrogels are susceptible to fatigue under prolonged loads (Bai et al., 2019). Fatigue of hydrogel is a molecular disease. Fatigue of tough hydrogels primarily stems from inelastic toughening mechanisms, in which the contribution of energy dissipation from the inelastic processes fades out over bitty by prolonged fatigue loading (Bai et al., 2017). Tough and fatigue-resistant hydrogels are synthesized based on elastic tougheners (Xiang et al., 2020). As for adhesion, because the polymer network is sparse and the water molecules barely carry load, the hydrogel often forms weak and unstable adhesion at the interface. The adhesion energy, measured as the energy needed to advance the interfacial crack per unit area, is typically on the order of 10^−1^ J/m^2^. Interfacial failure and therefore the loss of functionalities ensue with ease from the poor adhesion. Intensive efforts have been devoted recently to strengthening the adhesion between hydrogels and various materials, and adhesion energy of up to 1,000 J/m^2^ has been achieved (Yuk et al., 2016; Wirthl et al., 2017). Put together, however, it is conceivable that tough hydrogel adhesion based on inelastic tougheners is also prone to fatigue. Indeed, it has been reported that the adhesion energy between a calcium alginate-polyacrylamide hydrogel and a porcine skin is 580 J/m^2^ under monotonic load but curtails dramatically to 24 J/m^2^ under fatigue load (Ni et al., 2020). As a matter of course, fatigue-resistant adhesion can be realized by elastic tougheners such as long-chain polymers (Zhang et al., 2020). The polymer chain consists of repeated units of covalent bonds and is entropically elastic. When under load, all the covalent bonds are lengthened to the stretch limit elastically before fracture. When a single bond breaks, the elastic energy stored in the entire polymer chain dissipates. As such, the plausible verdict is that fatigue-resistant hydrogel adhesion depends on covalent bonds but negligibly on noncovalent interactions.

We note exceptions that fatigue-resistant hydrogel adhesion can be achieved based on physical interactions. Liu et al. have demonstrated robust adhesion, ~800 J/m^2^, between poly(vinyl alcohol) hydrogel and substrate under fatigue loading through the anchorage of ordered nanocrystalline domains with hydrogen bonds (Liu J. et al., 2020). We have recently reported a method to strengthen the adhesion between hydrogels by wrinkling (Li et al., 2021). For two hydrogel adherends and one hydrogel adhesive, appropriate hydrogen bonds and wrinkles are elaborately formed and regulated at the interface. The formation of wrinkles creates a tortuous path for crack propagation and an extended energy-dissipation zone to improve adhesion, transforming the once-adhesive failure to cohesive failure. On the other hand, the suppression of wrinkles can deactivate the adhesion-enhancement mechanism to facilitate effortless debonding, achieving on-demand benign detachment. Both hydrogen bonds and wrinkling are physical interactions. An immediate question emerges: does the physical adhesion suffer fatigue?

Symptoms of fatigue include the change in properties, such as elastic modulus, or the nucleation and growth of cracks. The former is called fatigue damage and the latter is called fatigue fracture. Both fatigue damage and fatigue fracture have been exhaustively studied for engineering materials such as metals, ceramics, polymers, and composites (Suresh and Ritchie, 1984; Suresh, 1998). Prominently distinct is the fact that a pre-crack is intentionally made in the study of fatigue fracture but not in the study of fatigue damage. In this work, we study the fatigue damage behaviors of a specific version of physical hydrogel adhesion. As previously described (Li et al., 2021), we synthesize polyacrylamide hydrogel as the adherend and poly(acrylic acid-co-acrylamide) hydrogel as the adhesive. Unlike what has been previously described, we form and regulate the profile of the wrinkle by biaxial prestretch and release. We carry out butt-joint tests to probe the adhesion performances. We show that, under monotonic loading, the peak stress of the samples with a wrinkled interface formed at λ_pre_ = 2 is enhanced by 17.7% as compared to an enhancement of 44.9% in that of the samples with a flat interface formed at λ_pre_ = 1. Under fatigue damage test, the samples with a wrinkled interface formed at λ_pre_ = 2 resist fatigue damage at a stress level of 70% peak stress, whereas the samples with a flat interface fail in ~9,000 cycles at a stress level of 63% peak stress. In addition, the endurance limit of the wrinkled-interface samples prepared at λ_pre_ = 2 is comparable to the peak stress of the flat-interface samples. Moreover, we find that the nearly perfectly elastic polyacrylamide hydrogel also suffers fatigue damage under the same experimental protocol and that its S-N curve is comparable to that of the interfacial fatigue in the wrinkled-interface samples prepared at λ_pre_ = 2, implying that the physical interaction–based interface can be as strong as the bulk of the covalently crosslinked hydrogel. We invoke the classical damage accumulation theory by tracking the evolution of the elastic modulus. When cohesive failure ensues, the evolutions of the elastic modulus of wrinkled-interface samples and hydrogel bulk, both in satisfactory agreements with the theory, are alike. We observe similar behaviors in different material systems with polyacrylamide hydrogels of different water contents. The presented observations suggest that fatigue-resistant adhesion between soft and wet materials such as hydrogels can also be engineered based on physical interactions.

## Experimental Section

### Materials

Monomers included acrylamide (AAm; Aladdin, A108465) and acrylic acid (AAc; Aladdin, A103526). Cross-linkers included N, N'-Methylenebis (acrylamide) (MBAA; Aladdin, M128783) and 3-(trimethoxysilyl) propyl methacrylate (TMSPMA, Aladdin, S111153). Initiators included α-ketoglutaric acid (Aladdin, K105571) and α, α'-Azodiisobutyramidine dihydrochloride (V50, ShangHai D&B Biological Science and Technology Co. Ltd.). All chemicals were purchased and used without further purification. Deionized water was used as the solvent for all solutions unless otherwise specified.

### Preparation of Polyacrylamide (PAAm) Hydrogel

Acrylamide powder (8.53 g) was first dissolved in deionized water (60 ml); then, 0.48 ml MBAA (0.1 mol L^−1^) and 1.2 ml α-ketoglutaric acid (0.1 mol L^−1^) were added. After vortex mixing for 1 min, the precursor was injected into a reaction mold, which was made of two parallel glass sheets (20 mm^2^ × 20 mm^2^) with an intervening silicone spacer (2-or 4-mm thick), and was subjected to UV light (365 nm, 15 W, Analytik Jena US, UVP XX-15BLB) for 1.5 h.

### Preparation of Dry Poly(Acrylic Acid-co-acrylamide) [(P(AAc-co-AAm)] Hydrogel Film

First, 11.25 ml AAc solution (2 mol L^−1^) and 3.75 ml AAm solution (2 mol L^−1^) were mixed in a reagent bottle; then, 28.5 μL TMSPMA and 300 μL V50 solution (0.1 mol L^−1^) were added to the mixture. After vortex mixing for 1 min, the solution was transferred into a plastic syringe and subjected to ultraviolet light for 4 min. Subsequently, the solution was dripped onto an acrylic disk (50 mm in diameter) and spin-coated at 800 rpm for 60 s. After that, the sample was immersed in hydrochloric acid solution (pH = 3.5) for 10 min to accelerate silane condensation. Finally, the sample was stored in an oven at 65°C for 4 h, followed by 12 h of exposure to the open air for thorough desiccation.

### Adhesion Procedure

The freshly prepared PAAm hydrogels were immediately stored in plastic bags to prevent dehydration. For the samples with a flat interface, a piece of dry P(AAc-co-AAm) hydrogel film was swelled in deionized water to equilibrium and then placed in between two pieces of PAAm hydrogels without prestretch, forming the PAAm/P(AAc-co-AAm)/PAAm laminate. For the samples with a wrinkled interface formed without prestretch (λ_pre_ = 1), the procedure is the same, except for the insertion of a dry P(AAc-co-AAm) film without pre-swelling. For the samples with a wrinkled interface formed with prestretch λ_pre_ = 2, two pieces of PAAm hydrogel were firstly prestretched to λ_pre_ = 2 and then were fixed; then, a piece of dry P(AAc-*co*-AAm) hydrogel film was placed on one of them. After quick placement of another prestretched PAAm hydrogel on the adhesive to form the PAAm/P(AAc-co-AAm)/PAAm laminate, the prestretch is released.

### Butt-Joint Test

The sandwiched laminate samples for the butt-joint tests were cut into circular sheets with a diameter of 30 mm using a laser cutter (Han's Laser, CMA0604-B-A). Each circular sample was glued to two acrylic sheets at the top and bottom, then to the substrate and the loading head of a mechanical tester. For monotonic loading, the top acrylic sheet was pulled vertically up by the machine (Instron 5966, 100-N load cell) at a constant speed of 15 mm/min. For the fatigue test, the machine (Instron ElectroPuls E3000, 250-N load cell) operated with a force-controlled triangular loading profile at a frequency of 5 Hz. After assembly, the samples were stored in sealed polyethylene bags and aged for 0.5 h before tests.

## Results and Discussion

**Figure 1:**
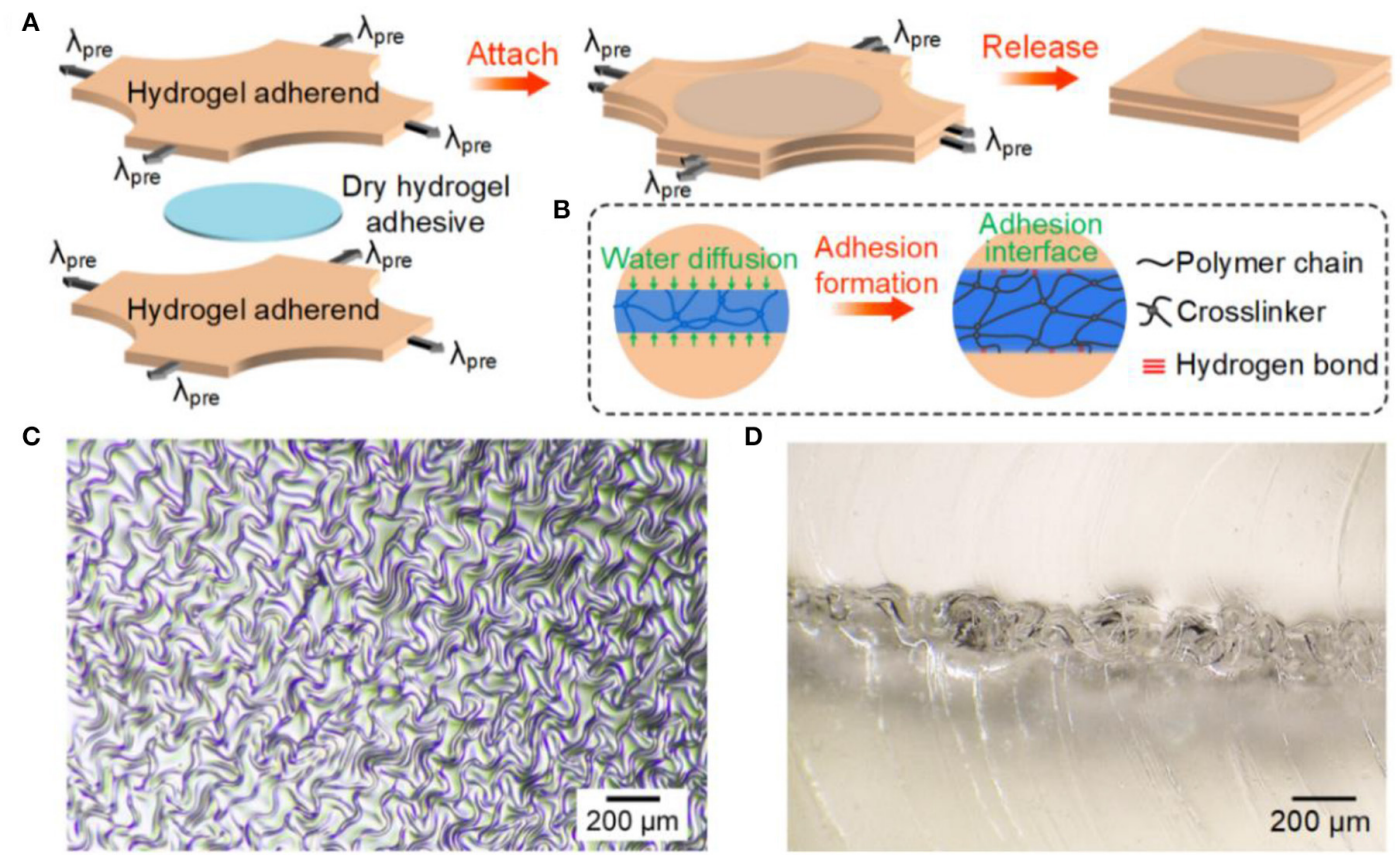
Preparation and characterization of samples. **(A)** Schematic of sample preparation. **(B)** Adhesion formation between adherend and adhesive. **(C)** Top view and **(D)** side view of wrinkles formed at λ_pre_ = 2. The adherend is PAAm and the adhesive is P(AAc-*co*-AAm) of molar fraction Φ_AAc_ = 0.75.

Physical hydrogel adhesion has various embodiments. Here, we selected a specific version based on hydrogen bonds and wrinkling (Li et al., 2021). We synthesized the adherend, PAAm hydrogel, by molding, and the adhesive, P(AAc-*co*-AAm) hydrogel, by spin-coating. We modified the P(AAc-*co*-AAm) copolymers with the silane coupling agent, 3-(trimethoxysilyl) propyl methacrylate (TMSPMA), such that the crosslinking process is decoupled from copolymerization and is able to be proceeded after spin-coat (Yao et al., 2019). Prior to adhesion, the P(AAc-*co*-AAm) hydrogel was thoroughly desiccated. Upon adhesion, hydrogen bonds formed between the carboxyl groups on P(AAc-*co*-AAm) and the amino groups on PAAm. The molar fraction of AAc in P(AAc-*co*-AAm) plays an important role in adhesion performance and was optimized at Φ_AAc_ = 0.75. In addition to hydrogen bonds, we further strengthened the adhesion by wrinkling. As depicted in Figure 1A, we prestretched two PAAm hydrogel adherends equibiaxially to λ_pre_ (defined as the prestretched length divided by original length), sandwiched a dry P(AAc-*co*-AAm) hydrogel adhesive to obtain the PAAm/P(AAc-*co*-AAm)/PAAm laminate, and then released the prestretch. Upon contact, the adhesive layer imbibed water from the adherends and underwent fast swelling. Adhesion sets in through the formation of hydrogen bonds (Figure 1B). After release, the two adherends sprung back to their original sizes, exerting in-plane compression on the swollen adhesive layer. As a result, the adhesive layer wrinkled (Figure 1C). Because the in-plane compression was equibiaxial, the wrinkles took random orientations. The cross-sectional view of a PAAm/P(AAc-*co*-AAm)/PAAm laminate prepared at λ_pre_ = 2 clearly demonstrated a mechanical interlocking structure at the interface (Figure 1D). It should be noted that the dry hydrogel adhesive was advantageous in eliminating interfacial water (Yuk et al., 2019), which has been known to weaken the adhesion (Rao et al., 2018), for rapid and robust hydrogel adhesion. The swelling strain would induce in-plane compression even without prestretch, whereas the wrinkling phenomenon was alleviated.

**Figure 2:**
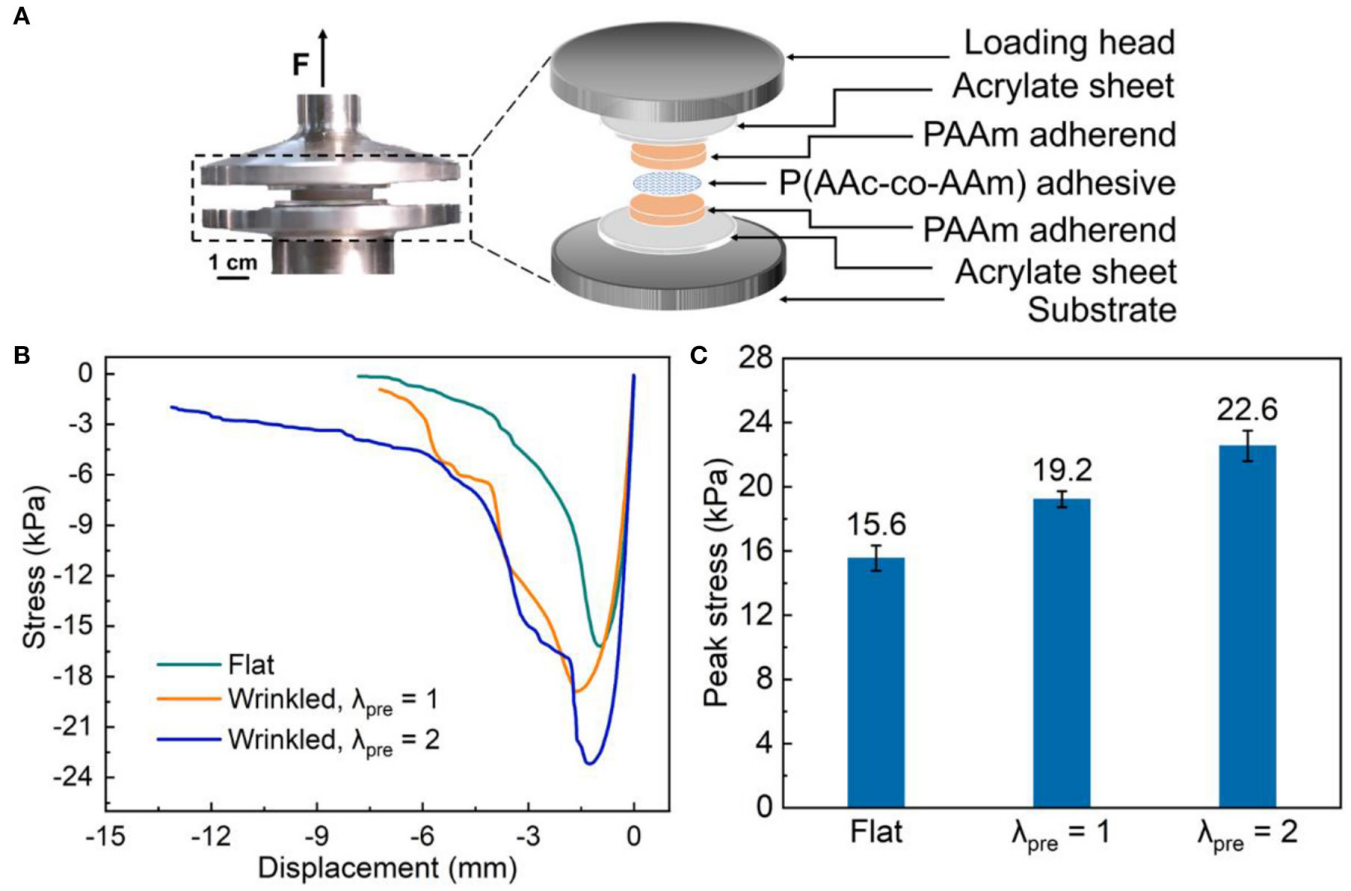
Adhesion under monotonic loading. **(A)** Photo and exploded schematic of the test setup. **(B)** Stress-displacement curves and **(C)** peak stresses with different interfacial morphologies. The error bars indicate the range of data, and at least three samples are tested for each case.

We firstly conducted monotonic butt-joint tests to probe the adhesion performance. The photo and the exploded schematic of the test setup are shown in Figure 2A. The laminate is about 4-mm thick and is cut into a circular disk of diameter 30 mm. Stress-displacement curves of samples with different interfacial morphologies under monotonic loading are collected in Figure 2B. Stress is calculated as force divided by the area of the adherend in the original state. As separation increases, stress rises, maximizes, and then goes down. The peak stress determines the strength of adhesion. The onset of stress drop is associated with visible local failures at the interface. The peak stress of wrinkled-interface samples prepared at λ_pre_ = 2 is 22.6 kPa, on average, was higher than that of the flat-interface samples, 15.6 kPa, and wrinkled-interface samples prepared at λ_pre_ = 1, 19.2 kPa, by 17.7 and 44.9%, respectively (Figure 2C). The improvement should be ascribed to the mechanical interlocking at the interface as well as the increase in effective adhesion area.

**Figure 3:**
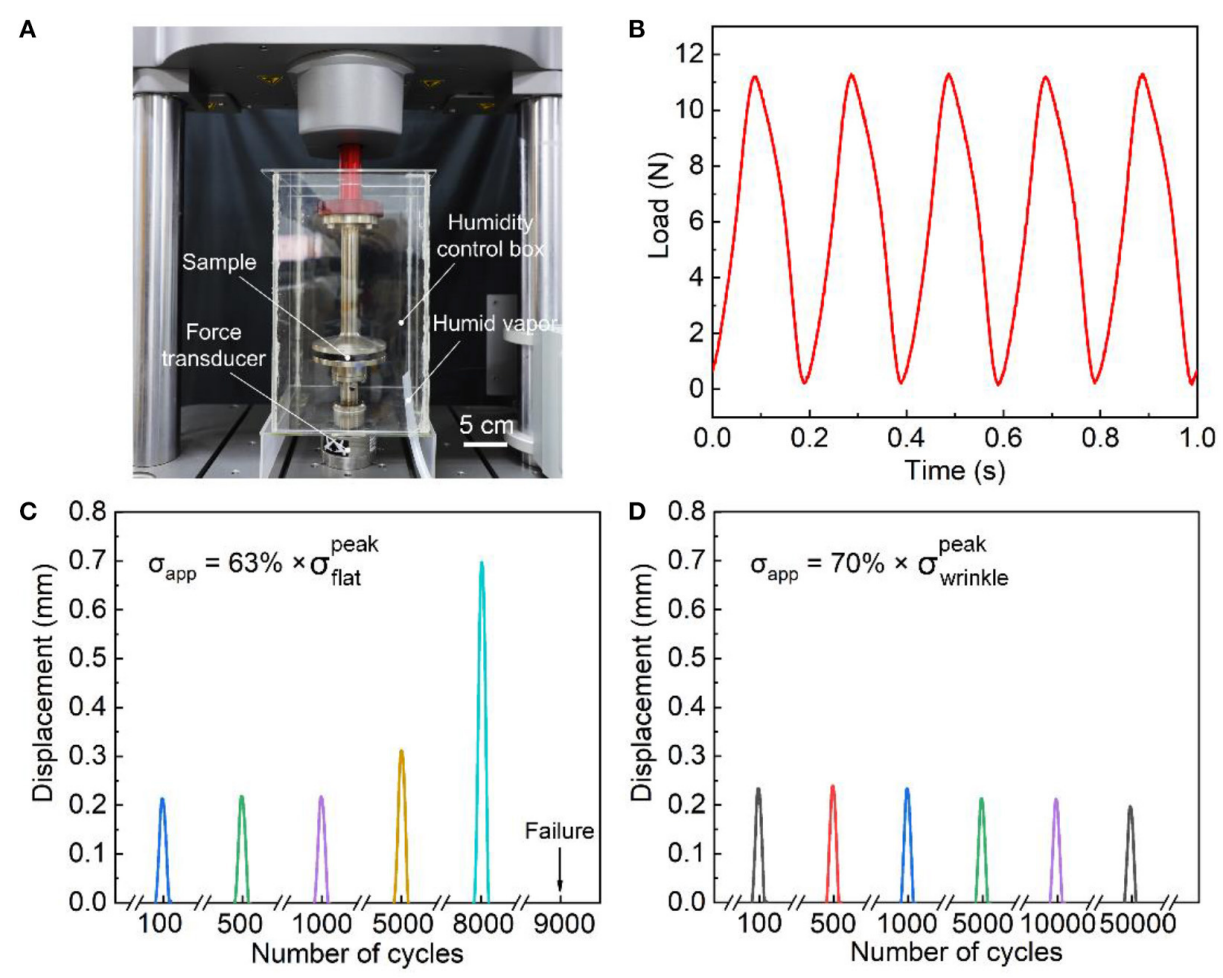
Fatigue damage test. **(A)** Experimental setup. **(B)** Representative loading profiles. Displacement vs. the number of cycles for the samples with **(C)** flat interface at 63% peak stress and **(D)** wrinkled interface formed at λ_pre_ = 2 at 70% peak stress.

We then conducted fatigue damage tests. Hereafter, we will focus on the wrinkled-interface samples of λ_pre_ = 2. To minimize dehydration of the hydrogels during the test, we seal the samples in a chamber of humidity control by pumping in vapor from a consumer humidifier (Bear Electric Appliance Co., Ltd) (Figure 3A). The weight loss of all samples was measured to be <2% after the test. We programmed a force-controlled triangular loading profile at a frequency of 5 Hz, as shown in Figure 3B. Although the triangular shape is distorted a bit, presumably due to inertia, the overall profiles maintained with considerable fidelity. We selected several stress levels lower than the static adhesion strength and recorded the displacement vs. the number of cycles until failure, which is the complete separation of the PAAm/P(AAc-*co*-AAm)/PAAm laminate. For the flat-interface samples, the displacement kept increasing with the number of cycles at a stress level of 63% peak stress. The sample failed after ~9,000 cycles (Figure 3C). The failure took place at the PAAm/P(AAc-*co*-AAm) interface. The strain rate enlarged as the sample crept, but the change was relatively small, e.g., from about 0.5/s at the 100th cycle to about 1.75/s at the 8,000th cycle. Since PAAm hydrogel is negligibly viscous and exhibits almost identical mechanical properties across three orders of magnitude of strain rates (Yang et al., 2019), the effect of viscoelasticity can be neglected. For the wrinkled-interface samples, the displacement under a stress level of 70% peak stress stayed almost constant up to 50,000 cycles (Figure 3D). The slight decrease in displacement was presumably caused by dehydration. The wrinkling not only enhanced the strength of adhesion but also strengthened the resistance to fatigue damage.

**Figure 4:**
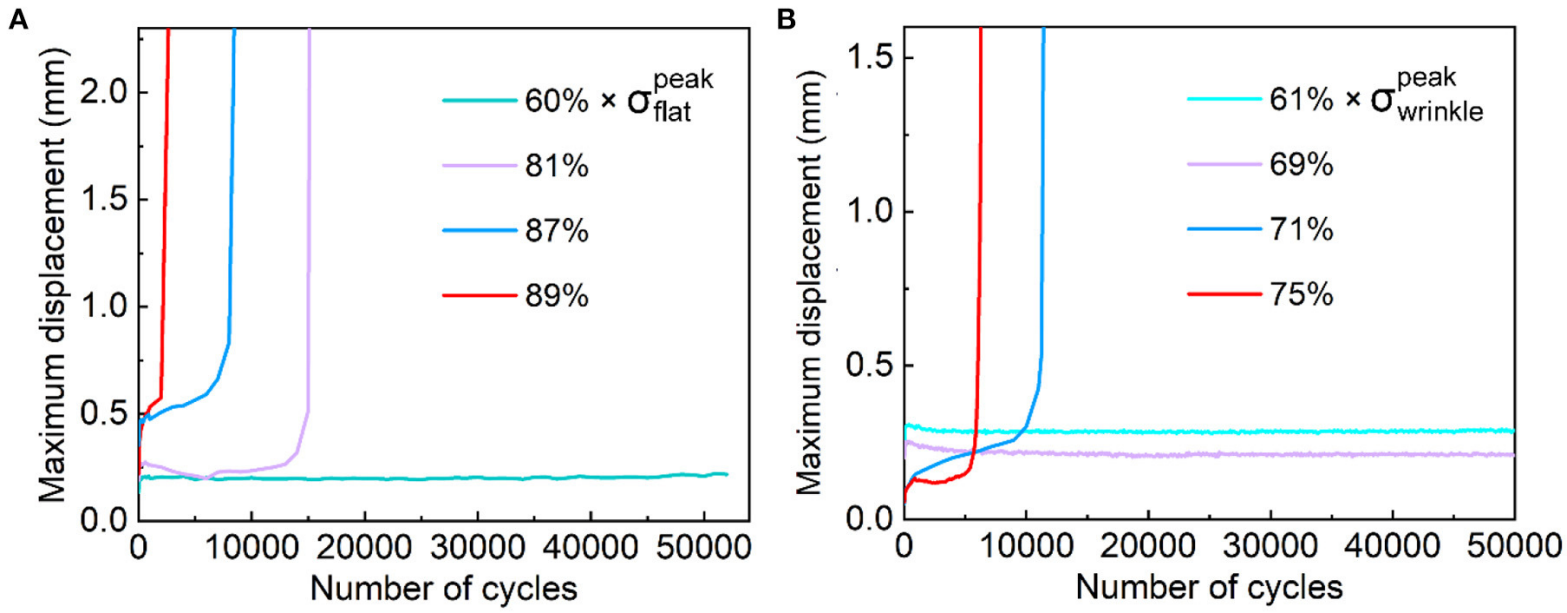
Maximum displacement during each load cycle vs. the number of cycles for the samples with **(A)** flat interfaces and **(B)** wrinkled interfaces formed at λ_pre_ = 2.

During the fatigue damage test, the maximum displacement during each cycle evolved with the number of cycles (Figure 4). After a period of initial damage accumulation, the maximum displacement rose sharply up at a certain critical loading cycle and the sample failed shortly afterward. The number of the critical loading cycle increased as the stress level decreased. For the flat-interface samples, the curves of maximum displacement vs. the number of cycles under the applied stresses of 60, 81, 87, and 89% of peak stress are plotted in Figure 4A. When the applied stress is 60% of peak stress, the maximum displacement increases slightly in the beginning, then flattens. We regarded the sample as fatigue damage-free if it survived 50,000 cycles in the experiments performed. In this sense, the flat-interface samples had a fatigue damage resistance of 60% peak stress. For the wrinkled-interface samples, the applied stresses were 61, 69, 71, and 75% of peak stress (Figure 4B). A fatigue-damage resistance of 69% peak stress was obtained.

**Figure 5:**
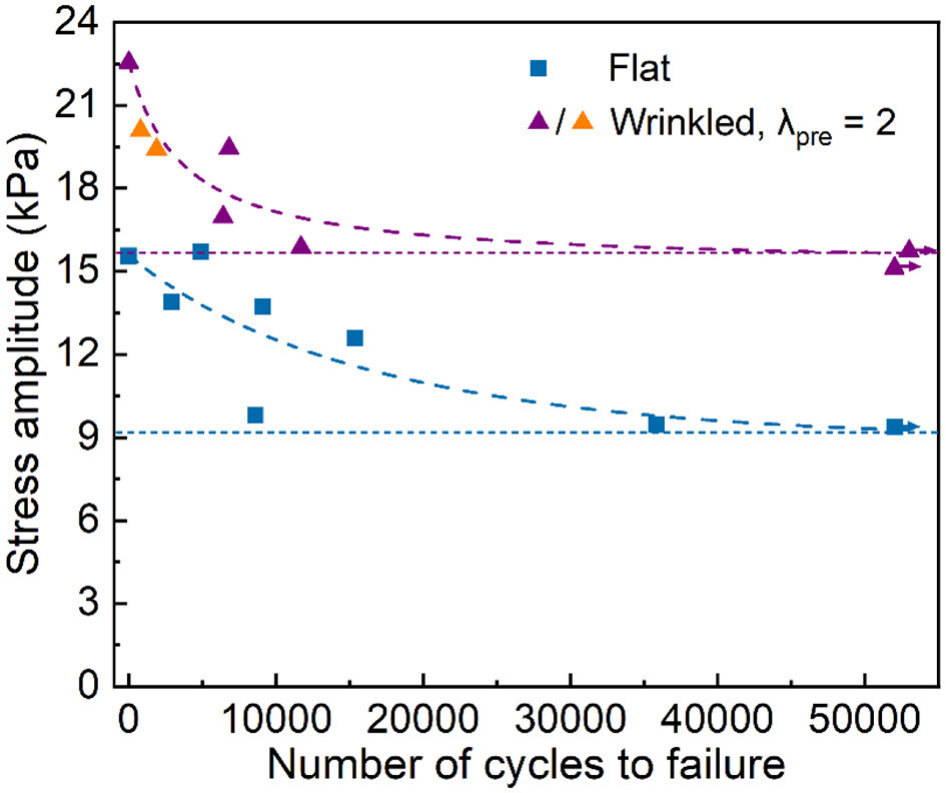
S-N curves for flat-interface samples and wrinkled-interface samples prepared at λ_pre_ = 2. Orange triangles indicate cohesive failure while all others exhibit interfacial damage. The horizontal dashed lines indicate the endurance limits and the curved dash lines are the guides for eye. Arrows mean that no failure occurs.

The plots of the S-N curves for the samples with flat/wrinkled interfaces are given in Figure 5. The amplitude of applied stress is given in the vertical axis and the number of cycles to failure is given in the horizontal axis. Each data point represents one fatigue damage test. As the stress amplitude decreases, the number of cycles to failure increases. The arrows associated with certain data points signify that the samples do not fail at the corresponding number of cycles. Following the previously mentioned definition, the fatigue damage resistance (or endurance limit) is 15.7 kPa for the wrinkled-interface samples and 9.37 kPa for the flat-interface samples. Notably, the endurance limit of the wrinkled-interface samples is comparable to the adhesion strength (i.e., peak stress) of the flat-interface samples. In addition to the mechanical interlocking at the interfaces and incremental effective adhesive area, the improvement of fatigue damage resistance of the wrinkled-interface samples should be attributed to the change in the orientation of the interface, which is once perpendicular to the applied load, after wrinkling such that the overall driving force of separation is mitigated to some extent. Furthermore, we observed that the wrinkled-interface samples at the stress amplitudes of 19.42 and 20.1 kPa persistently underwent cohesive failure (orange triangles in Figure 5), while other samples failed by adhesive failure (purple triangles and blue squares in Figure 5). Cohesive failure suggests that the physically strengthened interface could be at least as strong as the covalent bulk.

**Figure 6:**
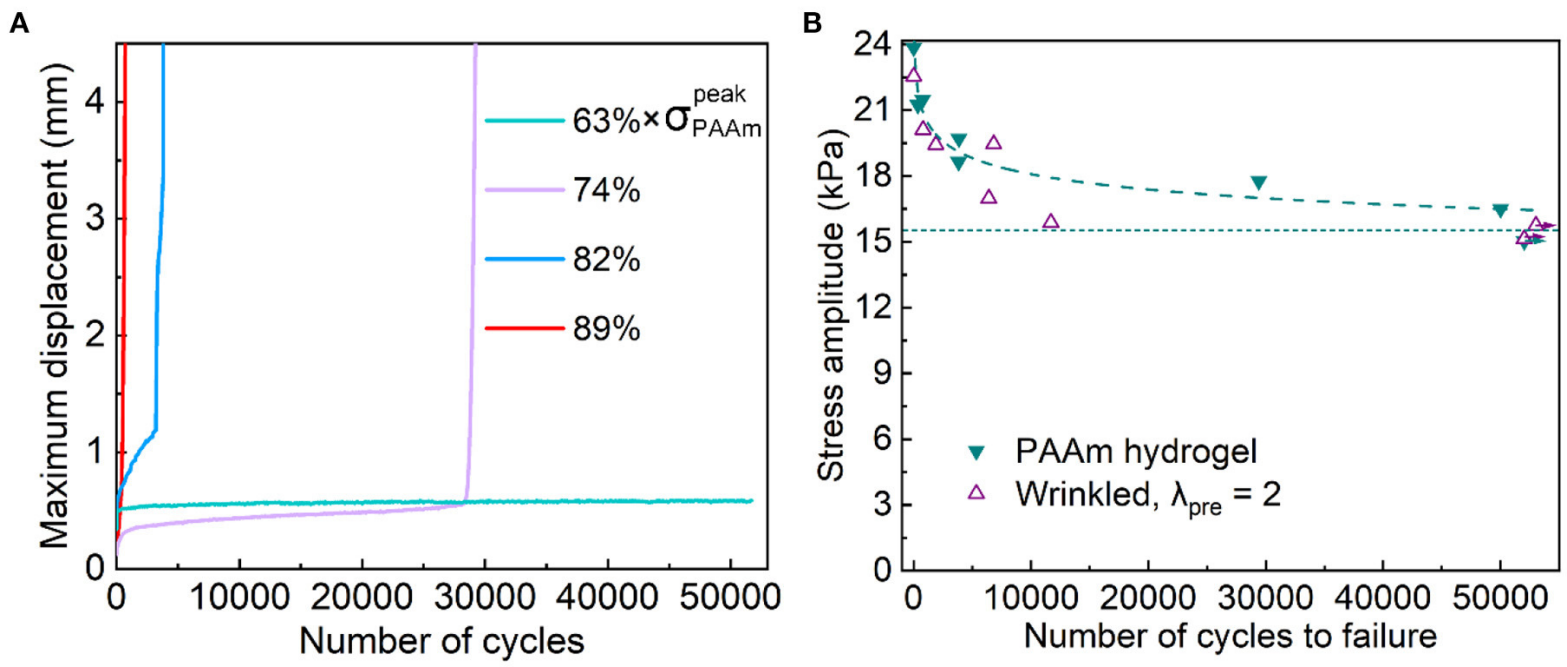
Fatigue damage tests for PAAm hydrogels. **(A)** Maximum displacement during each cycle vs. the number of cycles. **(B)** S-N curve. The data (hollow purple triangles) of the wrinkled interface formed at λ_pre_ = 2 are also plotted for comparison. The horizontal dashed line indicates endurance limit and the curved dash line is the guide for eye. Arrows mean that no failure occurs.

To ascertain the conjecture we arrived at, we further carried out the fatigue damage test for PAAm hydrogel using the same protocol. We synthesized cylindrical PAAm hydrogels of diameter 30 mm and height 4 mm. The curves of maximum displacement vs. the number of cycles at 63%, 74%, 82%, and 89% of peak stress are plotted in Figure 6A. The PAAm hydrogel resisted fatigue damage up to 63% peak stress. The S-N curve for the PAAm hydrogel is plotted in Figure 6B. The endurance limit of PAAm hydrogel was 15.1 kPa. The failure of PAAm hydrogels happened in the bulk but not at the junctions with the acrylate sheet.

The fatigue damage behavior of the PAAm hydrogel is interesting, provided that this particular hydrogel has been regarded as nearly perfectly elastic (Zhang et al., 2018) and widely used as a model material to study the mechanics of soft materials such as the growth of cracks in hydrogels under static (Tanaka et al., 2000), cyclic (Tang et al., 2017), and dynamic loads (Kolvin et al., 2018). In previous studies, however, the fatigue damage test of the PAAm hydrogel was conducted with constant maximum displacement (Bai et al., 2017). Under prolonged cyclic load with displacement control, the polymer network of a PAAm hydrogel might experience somewhat initial interior damages, such as chain scissions due to network imperfection (Yang et al., 2019; Lin and Zhao, 2020), but would stabilize as the external load diminishes. In the experiments performed in this study, the PAAm hydrogel was subjected to prolonged force-controlled cyclic loading. Under such a circumstance, as some polymer chains are broken, the load once borne by these broken chains will be transferred to the rest of the polymer chains, causing more scissions and more severe deformation of the polymer network. Consequently, the macroscopic deformation creeps from cycle to cycle and, eventually, the polymer network fractures at a certain critical point. A comprehensive understanding of the fatigue damage behavior of PAAm hydrogels with controlled load requires further study but is beyond the scope of the current paper.

Also plotted in Figure 6B are the data (hollow purple triangles) for the samples with a wrinkled interface. It is imperative to recall that PAAm hydrogel has a covalent polymer network while the PAAm/P(AAc-*co*-AAm)/PAAm laminate has purely physical interactions at the interfaces. Nevertheless, the two sets of samples have comparable life under the same load. While defects tend to nucleate at the interface and cause adhesive failure for the samples with a wrinkled interface, the S-N curve of the laminate sample is ultimately bounded by that of the PAAm hydrogel. Such encouraging results indicate that physical interactions can be engaged in engineering fatigue-resistant adhesion.

**Figure 7:**
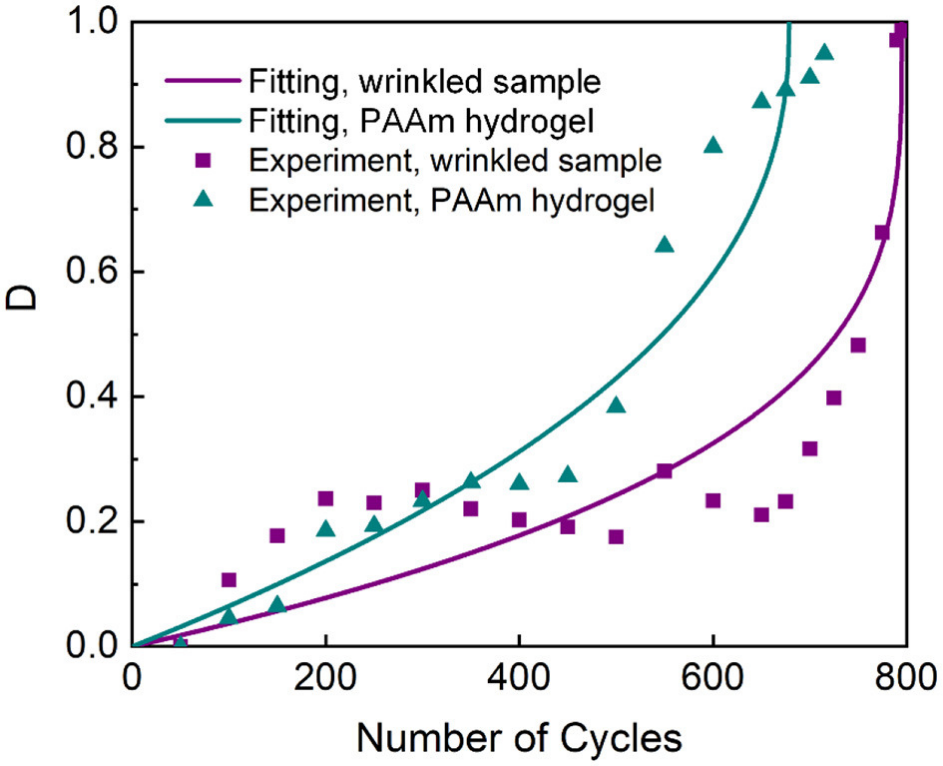
Damage variable *D* vs. the number of cycles for the wrinkled-interface samples prepared at λ_pre_ = 2 and PAAm hydrogels. The data for the wrinkled-interface sample are collected from a cohesive failure test. The applied stress is 89.4% peak stress. The curves are the best fit for the damage evolution model.

We invoke the classical damage accumulation theory to characterize the behaviors of fatigue damage. We define a fatigue damage variable *D* as *D* = 1 − *E*/*E*_0_, where *E* is the modulus at a certain number of cycles and *E*_0_ is the modulus at the 50th cycle. Here, we regard the PAAm/P(AAc-*co*-AAm)/PAAm laminate as an intact material. The modulus is calculated as the initial slop of the nominal stress-strain curve. The modulus at the 50th cycle is selected as *E*_0_ because the nominal stress-strain curves of the initial cycles are erratic. During the fatigue damage test, *D* firstly increases mildly from zero, and then goes up acutely to 1 upon fracture. We apply a fatigue damage evolution law to correlate the damage variable *D* to the number of cycles as follows (Abdel et al., 2010):

$$\begin{array}{l} N=C_{1}\left(1-{D_{m}}^{C_{2}}\right), \end{array}$$

where *N* is the number of cycles, *C*_1_ and *C*_2_ are fitting parameters, and *D*_*m*_ = 1 − *D*. As in most fatigue experiments, the data scatter enormously. Nevertheless, decent fitting results are obtained. As shown in Figure 7, the fitting of the equation to experimental data, collected from a cohesive failure test, gives $N=790\left(1-D_{m}^{6.2}\right)$ for the wrinkled-interface sample and $N=683\left(1-D_{m}^{2.44}\right)$ for the PAAm hydrogel under 89.4% peak stress, with *R*^2^ being 0.6777 and 0.9528, respectively. Despite the fitting errors, the raw data of the wrinkled samples and PAAm hydrogels demonstrate similar trends in the evolution of *D*, that is, an initial growth, then abeyance, and finally a rapid rise as the number of cycles increases. The similar trends in the evolution of *D* as well as the slower damage accumulation in the wrinkled-interface sample than that in the PAAm hydrogel further support the hypothesis that physically strengthened interfaces can be as strong as the covalent bulk.

**Figure 8:**
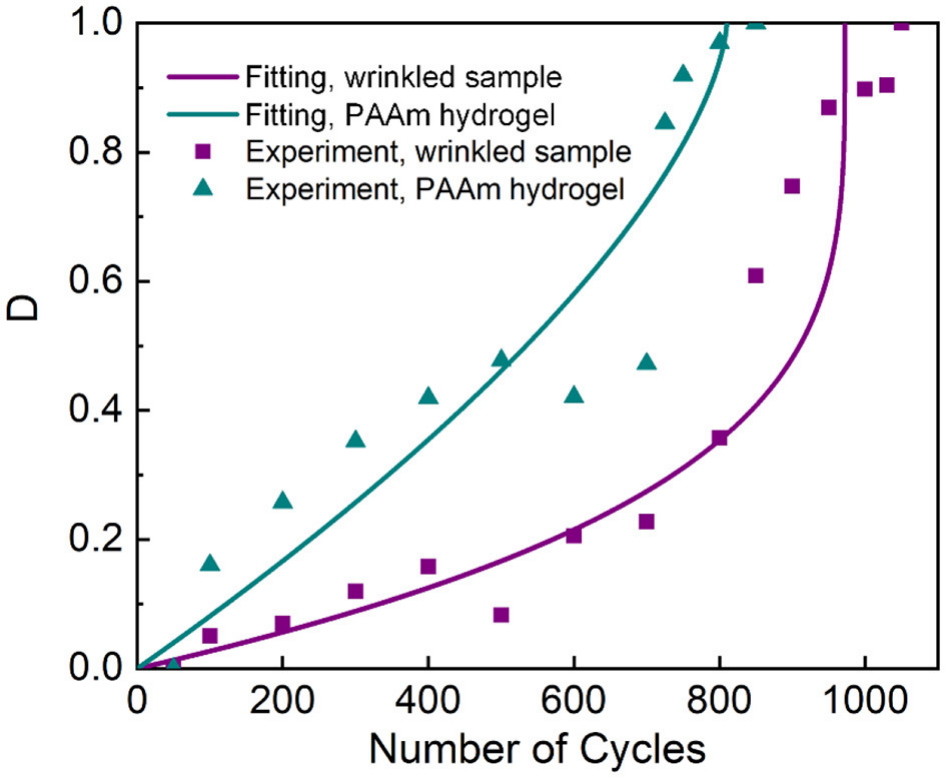
Damage variable *D* vs. the number of cycles for the wrinkled-interface samples prepared at λ_pre_ = 2 and PAAm hydrogel with higher water content. The data for the wrinkled-interface sample are collected from a cohesive failure test. The applied stress is 74.4% peak stress. The curves are the best fit to the damage evolution model.

Finally, we examine the feasibility of fatigue damage–resistant physical hydrogel adhesion on different material systems. We do so by pre-swelling PAAm hydrogels to increase the weight ratio by 23%, and then use the swollen PAAm hydrogels as the adherends for the PAAm/P(AAc-*co*-AAm)/PAAm laminate and the PAAm hydrogel bulk. We observe similar behaviors in fatigue damage tests. As shown in Figure 8, the fitting of the fatigue damage evolution law to the experimental data collected from the cohesive failure tests gives $N=972\left(1-D_{m}^{3.96}\right)$ for the wrinkled-interface sample and $N=810\left(1-D_{m}^{1.55}\right)$ for the PAAm hydrogel under 74.4% peak stress, with *R*^2^ being 0.9351 and 0.8814, respectively. These results further reinforce the viewpoint that physically strengthened hydrogel adhesions are capable of resisting fatigue damage.

## Conclusions

In summary, we have investigated and shown that physical hydrogel adhesion can resist fatigue damage by performing monotonic and cyclic butt-joint tests on PAAm/P(AAc-*co*-AAm)/PAAm laminates with different surface morphologies and PAAm hydrogel. We note that the hydrogen bonds are nanoscale physical interactions while wrinkles are microscale physical interactions. Without wrinkles, the adhesion has a low fatigue damage resistance. Without hydrogen bonds, the wrinkles cannot even be preserved. The synergy of multi-scale interactions is crucial for fatigue damage–resistant hydrogel adhesion. Such synergy has recently been revealed to be predominant in delaying fatigue fracture in polyampholyte hydrogels (Li X. et al., 2020). The findings of this work clarify that the fatigue damage of hydrogel adhesion can be resisted with the aid of physical interactions. Enormous immediate opportunities exist in exploring physical mechanisms of all kinds for the development of future soft and wet adhesion for a broad variety of engineering applications.

## Data Availability Statement

The original contributions presented in the study are included in the article/supplementary material, further inquiries can be directed to the corresponding author/s.

## Author Contributions

QLi, LW, and CY conceived the idea and wrote the manuscript. QLi and LW conducted the experiments and analyzed the data. WH provided suggestions and revised the manuscript. All authors discussed the results and commented on the manuscript.

## Conflict of Interest

The authors declare that the research was conducted in the absence of any commercial or financial relationships that could be construed as a potential conflict of interest.

## References

[B1] Abdel W. M. Hilmy I. Ashcroft I. A. Crocombe A. D. (2010). Evaluation of fatigue damage in adhesive bonding: part 1: bulk adhesive. J. Adhes. Sci. Technol. 24, 305–324. 10.1163/016942409X12508517390798

[B2] Acome E. Mitchell S. Morrissey T. Emmett M. B. Benjamin C. King M.. (2018). Hydraulically amplified self-healing electrostatic actuators with muscle-like performance. Science 359, 61–65. 10.1126/science.aao6139 29302008

[B3] Bai R. Yang J. Suo Z. (2019). Fatigue of hydrogels. Eur. J. Mech. A Solids 74, 337–370. 10.1016/j.euromechsol.2018.12.001

[B4] Bai R. Yang Q. Tang J. Morelle X. P. Vlassak J. Suo Z. (2017). Fatigue fracture of tough hydrogels. Extreme Mech. Lett. 15, 91–96. 10.1016/j.eml.2017.07.002

[B5] Calvert P. (2009). Hydrogels for soft machines. Adv. Mater. 21, 743–756. 10.1002/adma.200800534

[B6] Choi M. Choi J. W. Kim S. Nizamoglu S. Hahn S. K. Yun S. H. (2013). Light-guiding hydrogels for cell-based sensing and optogenetic synthesis *in vivo*. Nat. Photonics 7, 987–994. 10.1038/nphoton.2013.278 25346777 PMC4207089

[B7] Dubrovskii S. A. Afanas' Eva M. V. Lagutina M. A. Kazanskii K. S. (1990). Comprehensive characterization of superabsorbent polymer hydrogels. Polym. Bull. 24, 107–113. 10.1007/BF00298329

[B8] Gong J. Katsuyama Y. Kurokawa T. Osada Y. (2003). Double-network hydrogels with extremely high mechanical strength. Adv. Mater. 15, 1155–1158. 10.1002/adma.200304907

[B9] Keplinger C. Sun J. Y. Foo C. C. Rothemund P. Whitesides G. M. Suo Z. (2013). Stretchable, transparent, ionic conductors. Science 341, 984–987. 10.1126/science.1240228 23990555

[B10] Kim C. C. Lee H. H. Oh K. H. Sun J. Y. (2016). Highly stretchable, transparent ionic touch panel. Science 353, 682–687. 10.1126/science.aaf8810 27516597

[B11] King D. R. Sun T. Huang Y. Kurokawa T. Nonoyama T. Crosby A. J.. (2015). Extremely tough composites from fabric reinforced polyampholyte hydrogels. Mater. Horizons 2, 584–591. 10.1039/C5MH00127G

[B12] Kolvin I. Cohen G. Fineberg J. (2018). Topological defects govern crack front motion and facet formation on broken surfaces. Nat. Mater. 17, 140–144. 10.1038/nmat5008 29035358

[B13] Larson C. Peele B. Li S. Robinson S. Totaro M. Beccai L.. (2016). Highly stretchable electroluminescent skin for optical signaling and tactile sensing. Science 351, 1071–1074. 10.1126/science.aac5082 26941316

[B14] Lee Y. Song W. J. Sun J. Y. (2020). Hydrogel soft robotics. Mater. Today Phys. 15:100258. 10.1016/j.mtphys.2020.100258

[B15] Li J. Mooney D. J. (2016). Designing hydrogels for controlled drug delivery. Nat. Rev. Mater. 1, 1–17. 10.1038/natrevmats.2016.71 29657852 PMC5898614

[B16] Li Q. Xu Z. Ji S. Lv P. Li X. Hong W.. (2020). Kinetics-induced morphing of three-dimensional-printed gel structures based on geometric asymmetry. J. Appl. Mech. 87:071008. 10.1115/1.4046920

[B17] Li Q. Zhang P. Yang C. Duan H. Hong W. (2021). Switchable adhesion between hydrogels by wrinkling. Extreme Mech. Lett. 43:101193. 10.1016/j.eml.2021.101193

[B18] Li X. Cui K. Sun T. L. Meng L. Yu C. Li L.. (2020). Mesoscale bicontinuous networks in self-healing hydrogels delay fatigue fracture. Proc. Natl. Acad. Sci. U.S.A. 117, 7606–7612. 10.1073/pnas.2000189117 32209673 PMC7149489

[B19] Lin S. Zhao X. (2020). Fracture of polymer networks with diverse topological defects. Phys. Rev. E 102:052503. 10.1103/PhysRevE.102.052503 33327130 PMC8019060

[B20] Liu J. Lin S. Liu X. Qin Z. Yang Y. Zang J.. (2020). Fatigue-resistant adhesion of hydrogels. Nat. Commun. 11:1071. 10.1038/s41467-020-14871-3 32103027 PMC7044439

[B21] Liu X. Liu J. Lin S. Zhao X. (2020). Hydrogel machines. Mater. Today 36, 102–124. 10.1016/j.mattod.2019.12.026

[B22] Nam S. Mooney D. (2021). Polymeric tissue adhesives. Chem. Rev. 10.1021/acs.chemrev.0c00798. [Epub ahead of print]. 33507740

[B23] Ni X. Chen C. Li J. (2020). Interfacial fatigue fracture of tissue adhesive hydrogels. Extreme Mech. Lett. 34:100601. 10.1016/j.eml.2019.100601

[B24] Pu X. Guo H. Chen J. Wang X. Xi Y. Hu C.. (2017). Eye motion triggered self-powered mechnosensational communication system using triboelectric nanogenerator. Sci. Adv. 3:e1700694. 10.1126/sciadv.1700694 28782029 PMC5533541

[B25] Rao P. Sun T. Chen L. Takahashi R. Shinohara G. Guo H.. (2018). Tough hydrogels with fast, strong, and reversible underwater adhesion based on a multiscale design. Adv. Mater. 30:1801884. 10.1002/adma.201801884 29939425

[B26] Schroeder T. B. Guha A. Lamoureux A. VanRenterghem G. Sept D. Shtein M.. (2017). An electric-eel-inspired soft power source from stacked hydrogels. Nature 552, 214–218. 10.1038/nature24670 29239354 PMC6436395

[B27] Sun J. Y. Keplinger C. Whitesides G. M. Suo Z. (2014). Ionic skin. Adv. Mater. 26, 7608–7614. 10.1002/adma.201403441 25355528

[B28] Sun J. Y. Zhao X. Illeperuma W. R. Chaudhuri O. Oh K. H. Mooney D. J.. (2012). Highly stretchable and tough hydrogels. Nature 489, 133–136. 10.1038/nature11409 22955625 PMC3642868

[B29] Suresh S. Ritchie R. (1984). Propagation of short fatigue cracks. Int. Met. Rev. 29, 445–475. 10.1179/imr.1984.29.1.445

[B30] Suresh S. (1998). Fatigue of Materials. Cambridge: Cambridge University Press. 10.1017/CBO9780511806575

[B31] Tanaka Y. Fukao K. Miyamoto Y. (2000). Fracture energy of gels. Eur. Phys. J. E 3, 395–401. 10.1007/s101890070010

[B32] Tang J. Li J. Vlassak J. J. Suo Z. (2017). Fatigue fracture of hydrogels. Extreme Mech. Lett. 10, 24–31. 10.1016/j.eml.2016.09.010

[B33] Thiele J. Ma Y. Bruekers S. M. Ma S. Huck W. (2014). 25th anniversary article: designer hydrogels for cell cultures: a materials selection guide. Adv. Mater. 26, 125–148. 10.1002/adma.201302958 24227691

[B34] Wichterle O. Lim D. (1960). Hydrophilic gels for biological use. Nature 185, 117–118. 10.1038/185117a0

[B35] Wirthl D. Pichler R. Drack M. Kettlguber G. Moser R. Gerstmayr R.. (2017). Instant tough bonding of hydrogels for soft machines and electronics. Sci. Adv. 3:e1700053. 10.1126/sciadv.1700053 28691092 PMC5479648

[B36] Xiang C. Wang Z. Yang C. Yao X. Wang Y. Suo Z. (2020). Stretchable and fatigue-resistant materials. Mater. Today 34, 7–16. 10.1016/j.mattod.2019.08.009

[B37] Yang C. Chen B. Lu J. Yang J. Zhou J. Chen Y.. (2015). Ionic cable. Extreme Mech. Lett. 3, 59–65. 10.1016/j.eml.2015.03.001

[B38] Yang C. Chen B. Zhou J. Chen Y. Suo Z. (2016). Electroluminescence of giant stretchability. Adv. Mater. 28, 4480–4484. 10.1002/adma.201504031 26610277

[B39] Yang C. Cheng S. Yao X. Nian G. Liu Q. Suo Z. (2020). Ionotronic luminescent fibers, fabrics, and other configurations. Adv. Mater. 32:2005545. 10.1002/adma.202005545 33089568

[B40] Yang C. Suo Z. (2018). Hydrogel ionotronics. Nat. Rev. Mater. 3:125. 10.1038/s41578-018-0018-7

[B41] Yang C. Yin T. Suo Z. (2019). Polyacrylamide hydrogels. I. Network imperfection. J. Mech. Phys. Solids 131, 43–55. 10.1016/j.jmps.2019.06.018

[B42] Yang C. Zhou S. Shian S. Clarke D. R. Suo Z. (2017). Organic liquid-crystal devices based on ionic conductors. Mater. Horizons 4, 1102–1109. 10.1039/C7MH00345E

[B43] Yao X. Liu J. Yang C. Yang X. Wei J. Xia Y.. (2019). Hydrogel paint. Adv. Mater. 31:1903062. 10.1002/adma.201903062 31379064

[B44] Yuk H. Varela C. E. Nabzdyk C. S. Mao X. Padera R. F. Roche E. T.. (2019). Dry double-sided tape for adhesion of wet tissues and devices. Nature 575, 169–174. 10.1038/s41586-019-1710-5 31666696

[B45] Yuk H. Zhang T. Lin S. Parada G. A. Zhao X. (2016). Tough bonding of hydrogels to diverse non-porous surfaces. Nat. Mater. 15, 190–196. 10.1038/nmat4463 26552058 PMC4762474

[B46] Zhang E. Bai R. Morelle X. P. Suo Z. (2018). Fatigue fracture of nearly elastic hydrogels. Soft Matter 14, 3563–3571. 10.1039/C8SM00460A 29682668

[B47] Zhang W. Gao Y. Yang H. Suo Z. Lu T. (2020). Fatigue-resistant adhesion I. Long-chain polymers as elastic dissipaters. Extreme Mech. Lett. 39:100813. 10.1016/j.eml.2020.100813

